# Recruitment of renal functional reserve by intravenous amino acid loading in a sheep model of cardiopulmonary bypass

**DOI:** 10.1186/s40635-025-00774-4

**Published:** 2025-07-10

**Authors:** Taku Furukawa, Alemayehu H. Jufar, Clive N. May, Roger G. Evans, Andrew D. Cochrane, Bruno Marino, Peter R. McCall, Sally G. Hood, Ian E. Birchall, Jaishankar Raman, Pei Chen Connie Ow, Anton Trask-Marino, Lachlan F. Miles, Rinaldo Bellomo, Yugeesh R. Lankadeva

**Affiliations:** 1 Preclinical Critical Care Unit, Florey Institute of Neuroscience and Mental Health, University of Melbourne, Melbourne, Australia; 2 Department of Critical Care, Melbourne Medical School, University of Melbourne, Melbourne, Australia; 3 Cardiovascular Disease Program, Biomedicine Discovery Institute and Department of Physiology, Monash University, Melbourne, Australia; 4 Cellsaving and Perfusion Resources, Melbourne, Australia; 5 Department of Anaesthesia, Austin Health, Heidelberg, Australia; 6 Faculty of Medicine, Dentistry and Health Sciences, University of Melbourne, Melbourne, Australia; 7 Department of Intensive Care, Austin Health, Heidelberg, Australia; 8 Australian and New Zealand Intensive Care Research Centre, Monash University, Melbourne, Australia; 9 Department of Intensive Care, Royal Melbourne Hospital, Melbourne, Australia

**Keywords:** Cardiopulmonary bypass, Cardiac surgery, Renal functional reserve, Acute kidney injury, Acute kidney disease, Chronic kidney disease, Amino acids

## Abstract

**Background:**

Cardiopulmonary bypass (CPB) may decrease the renal functional reserve (RFR). However, the temporal changes in RFR after during the recovery period after CPB remains unknown. We assessed RFR before and then weekly after CPB over four weeks following CPB in non-anaesthetised sheep.

**Methods:**

In 10 Merino ewes, amino acids were infused before CPB and weekly for four weeks to assess RFR. At each assessment, we measured renal blood flow (RBF), renal oxygen delivery (RDO_2_), creatinine clearance and medullary and cortical oxygenation. Histological assessment was performed at 4 weeks.

**Results:**

Before CPB, amino acid infusion increased RBF from (mean ± SD) 6.60 ± 1.64 to 8.56 ± 1.80 mL/kg/min, and RDO_2_ from 0.80 ± 0.28 to 1.12 ± 0.37 mL O_2_/kg/min. These renal macro-circulatory responses remained consistent across all weekly assessments after CPB. Amino acid infusion also increased creatinine clearance (from 62.5 ± 15.0 to 110 ± 30.6 mL/h pre-CPB) throughout the study period. RFR remained unchanged over time (P = 0.53). However, compared with pre-CPB values, medullary (33.9 ± 9.0 pre-CPB to 15.1 ± 13.2 mmHg at 4 weeks, P = 0.0068) and cortical tissue PO_2_ (46.0 ± 14.2 to 17.2 ± 6.5 mmHg, P = 0.0029) decreased over time. Furthermore, the response of the medullary (but not cortical) PO₂ to amino acid infusion changed over time (P = 0.0064). While medullary PO₂ did not change in response to amino acid infusion pre-CPB and at one week after CPB, it appeared to fall from two weeks thereafter (P = 0.039 and 0.091 at weeks 2 and 3, respectively). Despite preserved RFR, sheep exposed to CPB showed greater peritubular inflammation, interstitial fibrosis and tubular casts compared with healthy controls (P = 0.007, 0.021, 0.007, respectively).

**Conclusions:**

In this large mammalian model of CPB, weekly amino acid administration consistently recruited RFR over four weeks, despite the presence of histological injury. However, it was associated with the development of renal medullary hypoxia after two weeks. These findings highlight the complexity of the pathophysiological response of the kidney to CPB.

**Supplementary Information:**

The online version contains supplementary material available at 10.1186/s40635-025-00774-4.

## Introduction

Cardiopulmonary bypass (CPB) is injurious to the kidneys [1] and increases the risk of acute kidney injury (AKI) [2] and need for renal replacement therapy [3]. AKI is also associated with increased risk of chronic kidney disease (CKD) [4, 5], reduced quality of life, and higher health care costs [6, 7]. Moreover, even in the absence of AKI, one in ten patients exposed to CPB will develop significant (> 20%) loss of estimated glomerular filtration rate (GFR) within 12 months. [8]

However, even though most patients have no identifiable increase in serum creatinine, there may still be effects of CPB on renal function that are biologically and physiologically important. In particular, patients exposed to CPB may lose renal functional reserve (RFR) despite no identifiable changes in serum creatinine [9]. RFR is the ability of the kidneys to increase GFR in response to physiological stimuli, such as an oral protein load or amino acid (AA) infusion [10, 11]. Preserving RFR is important because its loss exposes the residual functioning nephrons to hyperfiltration, which is one of the pathophysiological mechanisms of progressive CKD. Assessment of RFR before and after CPB, therefore, has the potential to detect a post-CPB loss of renal function that is not revealed by creatinine measurements.

Accordingly, we developed a sheep model of CPB with a four-week postoperative recovery period, with monitoring of systemic haemodynamics and renal macro- and micro-circulatory function [1]. Using this model, we aimed to test the hypothesis that RFR is reduced following CPB. In addition, in kidneys recovering from CPB, we aimed to assess whether, independent on any effect on GFR, repeated testing for RFR by AA loading is associated with effects on medullary and cortical oxygenation like those observed before CPB.

## Methods

All experimental procedures were approved by the Animal Ethics Committee of the Florey Institute of Neuroscience and Mental Health (approval number 18–119-FINMH). All data are reported according to the Animal Research: Reporting of In Vivo Experiments (ARRIVE 2.0) guidelines. [12]

This study is a sub-study of recently published work [1]. Fifteen healthy, adult Merino ewes (1.5–2.0 years of age) were included in this study. Ten sheep, with a mean body weight of 37.1 kg (standard deviation [SD] 3.0), underwent the previously described CPB protocol [1]. An additional five healthy animals were included as a control group for histological analysis. Histological assessments were conducted by a pathologist who was blinded to the treatment allocation.

### Surgical preparation and CPB

Prior to experimentation, animals were acclimatised to the laboratory environment for one week. They were housed individually in metabolic cages with unrestricted access to 5 L of water and 800 g of oaten chaff per day.

Each animal underwent two surgical preparations, followed by an experimental CPB procedure, as described previously [1]. In the first surgical procedure, bilateral carotid arterial loops were constructed to provide long-term vascular access for arterial cannulation and blood sampling [13]. A recovery period of 3–4 weeks was allowed for the arteries to heal and become fully accessible. After this recovery period, a second surgical procedure was performed to: (1) place a transit-time flow probe (4 mm, Transonic Systems, New York, United States) around the left renal artery; (2) insert custom-made combination fiber-optic probes (Oxford Optronix, Abingdon, United Kingdom) into the renal medulla and cortex for measurement of renal tissue oxygen tension (PO_2_) and temperature; (3) insert a urinary bladder catheter (Foley size 14 French, 30 ml; Euromedical). The positions of the probe tips within the renal medulla and cortex were confirmed postmortem.

After at least 4 days recovery, the sheep were anaesthetised and a transit-time flow probe (20 mm; Transonic Systems) was placed around the pulmonary artery for measurement of cardiac output. Sheep then underwent mildly hypothermic CPB with an aortic cross clamp applied for 2 h, followed by recovery, as detailed previously [1]. A non-pulsatile flow of 2.4 L/min/m [2] (body surface area calculated according to Bennett [14]), mean arterial pressure (MAP) of 65–75 mmHg and body temperature of 36 °C (the basal body temperature in sheep being 39–40 °C) were targeted, consistent with current best practice recommendations in humans [15].Myocardial protection was achieved with antegrade blood cardioplegia. Following weaning from CPB and chest closure, anaesthesia was discontinued, and the trachea was extubated when clinically appropriate. The sheep were then transferred to metabolic cages for postoperative monitoring. Once fully conscious, they were given unrestricted access to food and water and were monitored for 4 weeks.

### Experimental protocol for RFR assessment

**Fig. 1:**
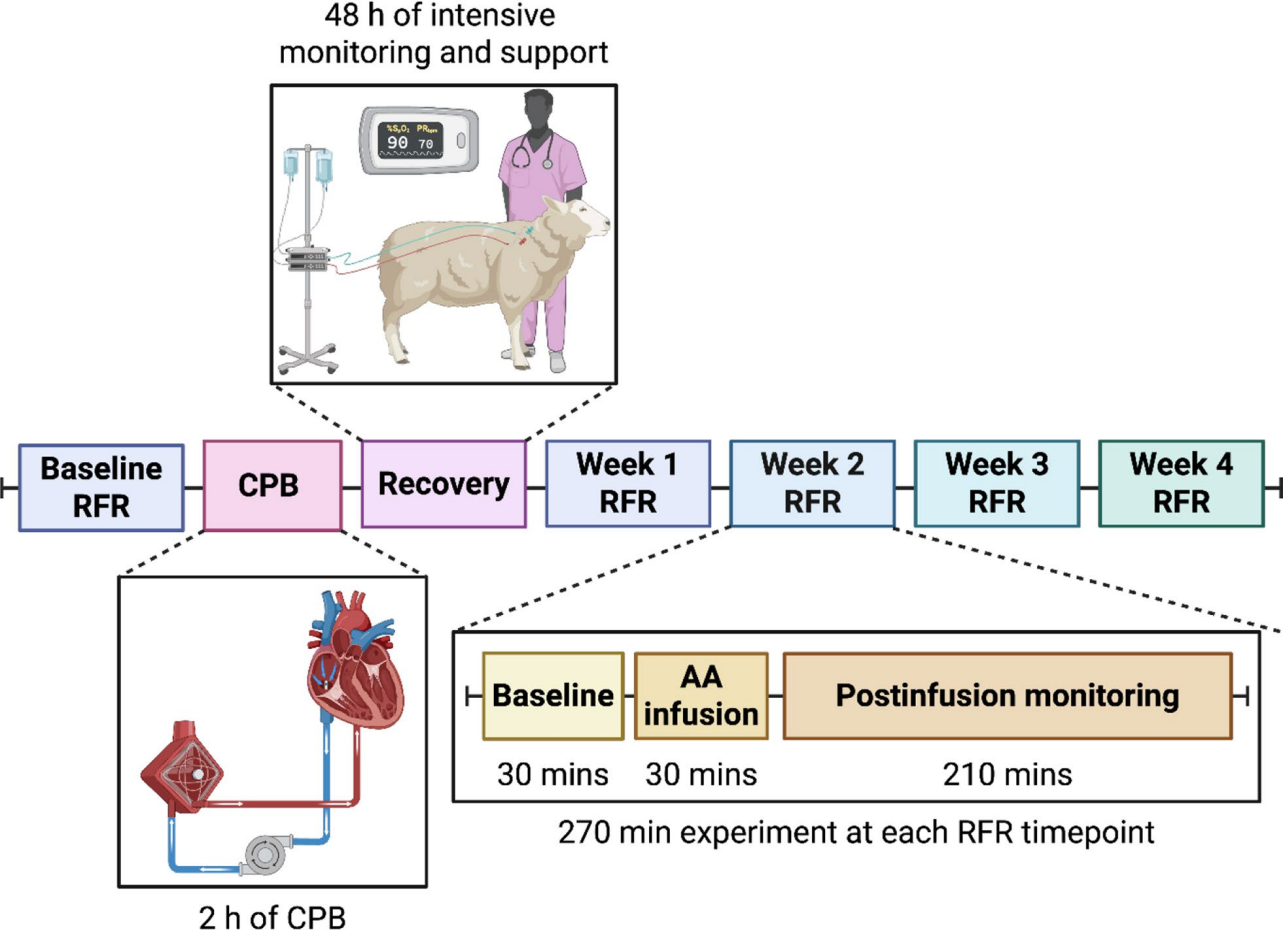
Schematic description of the study. Abbreviations: AA, amino acids; CPB, cardiopulmonary bypass; RFR, renal functional reserve

The RFR experiments were conducted two days before CPB and 1, 2, 3 and 4 weeks after CPB, while the sheep were conscious and unrestricted in individual metabolic cages. After an initial 30-min baseline period, a proprietary mixture of L-amino acids (500 mL of 10% Synthamin^®^ 17, 50 g/500 mL, Electrolyte-Free; Baxter Healthcare) was administered intravenously over the second 30-min period at a rate of 1000 mL/h [16]. This delivered a median dose of AAs of 1.46 g/kg. This was followed by additional a 3.5-h experimental period to assess renal functional and physiological responses (Fig. 1).

### Experimental measurements

Blood and urine samples were collected every 30 min for measurement of creatinine and sodium, with arterial blood samples for oximetry and biochemical analysis (ABL system 625, Radiometer Medical, Copenhagen, Denmark). Creatinine clearance, as an estimate of GFR, was calculated as the product of the urinary concentration of creatinine and urine flow divided by the plasma concentration of creatinine. RFR was determined as the difference between the maximum creatinine clearance during the experimental periods and the baseline (resting) clearance from the first 30-min period. Arterial pressure, cardiac output, renal blood flow (RBF), renal medullary and cortical PO_2_, and temperature were continuously recorded as previously described [17]. Medullary and cortical PO₂ values were averaged over 1.5 h from the start of AA infusion and compared with baseline levels. Renal vascular conductance (RVC) was calculated by dividing RBF by the product of MAP and body weight. Renal oxygen delivery (RDO_2_) was determined as the product of RBF and arterial oxygen content.

### Histological analysis

At the end of the experiment, animals were humanely euthanised with intravenous sodium pentobarbital (100 mg/kg; Lethobarb, Virbac, VIC, Australia) and the kidneys were immediately collected for histological analysis. The kidneys were sliced transversely and fixed in 10% neutral buffered formalin for 14 days. Three representative segments, including the medulla, cortex, and papilla, were paraffin-embedded and sectioned for analysis. Histological analysis was performed using haematoxylin and eosin, periodic acid-Schiff, and Masson’s trichrome stains by a pathologist blinded to treatment allocation. Acute tubular necrosis was assessed based on tubular dilatation, epithelial thinning, regenerative changes, and vacuolation. Interstitial tissue was evaluated for peritubular inflammatory infiltrates and fibrosis. Glomeruli were assessed for the changes of focal and segmental hyalinosis and sclerosis, capillary loop thrombi and mesangial proliferation. Changes were graded semi-quantitatively as 0 (no change), + (mild focal, 0–10%), + + (moderate focal, 10–50%), and + + + (severe diffuse, ≥ 50%), as previously described. [1, 18]

### Statistical analysis

Statistical analyses were performed using GraphPad Prism® for Windows, version 10 (GraphPad Software, Boston, MA, USA). Data are presented as mean (SD) or median [interquartile range, IQR], as appropriate. For paired comparisons (e.g., resting vs. post–AA infusion), paired t-tests were used, with the Holm–Šidák method applied for multiple comparisons. For post-CPB measurements, mixed-effects modelling was applied with a Greenhouse–Geisser correction for the main effect of time. For variables that significantly violated normality according to the Shapiro–Wilk test, such as urine output, Friedman’s test was used. Histological data were analysed using Fisher’s exact test for categorical data and the Mann–Whitney U test for ordinal data, as described previously [1, 18]. Two-sided p-values ≤ 0.05 were considered statistically significant.

## Results

All ten animals were successfully weaned off CPB. Eight animals completed the full 4-week experimental protocol and were included in the histological analysis. Two animals were euthanised between weeks 2 and 4 due to malfunction of a carotid arterial loop, which was unrelated to the study measurements. Therefore, the data collected from these animals prior to euthanasia were included in the analysis. Among the ten animals, four (40%) met the KDIGO criteria [19] for stage 1 AKI based on urine output.

### Systemic haemodynamics

The administration of an AA load resulted in a transient increase in MAP at 30 min. This was followed by a decrease (approximately 10 mmHg) to values below baseline, lasting several hours during each RFR assessment from pre-CPB to week 4 (Figure S1a-e). In contrast, cardiac output increased transiently in the first 1 to 1.5 h in response to AA infusion (Figure S1f-i) and then returned to baseline.

### Renal haemodynamics

**Fig. 2:**
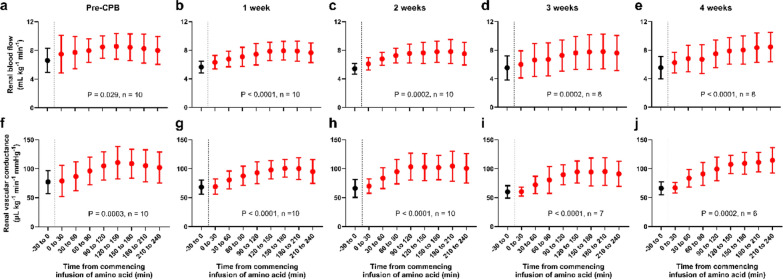
Changes in renal haemodynamics following amino acid infusion. (**a**–**e**) Renal blood flow; (**f**–**j**) renal vascular conductance. Amino acids were infused over the 0–30 min period. Sample sizes are indicated in each panel, as some data were unavailable due to equipment failure or test subject loss. P-values were obtained using a mixed-effects model with a Greenhouse–Geisser correction applied to the main effect of time. Data are presented as mean ± SD. *CPB* cardiopulmonary bypass

The administration of AA to recruit RFR induced a sustained increase in RBF, which persisted for 4 h (Fig. 2a–e). This response was consistent in magnitude and observed across all assessments. The increase in RBF was associated with an increase in renal vascular conductance, consistently observed across all assessments, indicating renal vasodilation (Fig. 2f–j). Consistent with increased RBF, renal oxygen delivery significantly increased following AA infusion (Figure S2).

### Renal tissue oxygenation

**Fig. 3:**
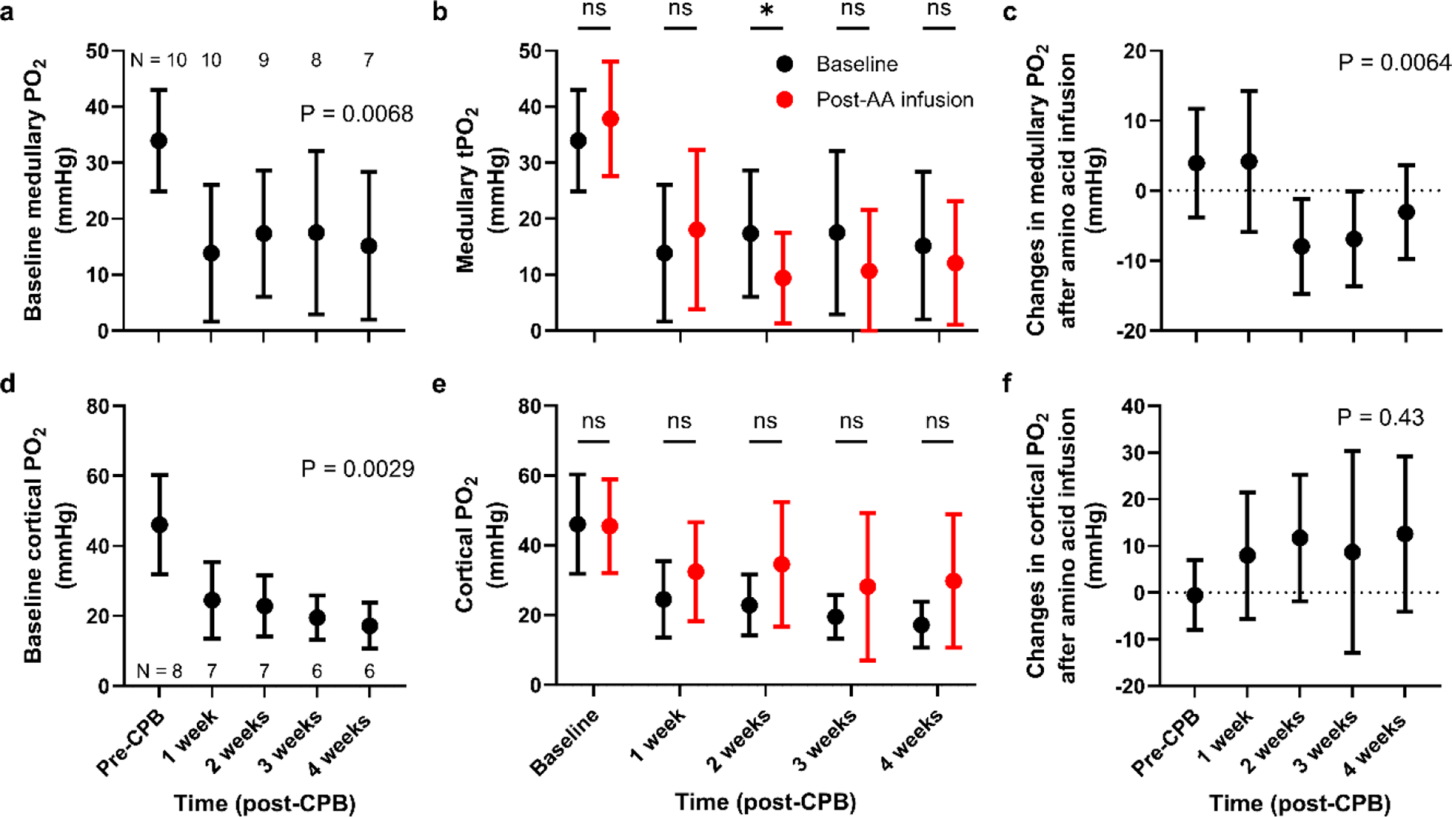
Renal microcirculation. (**a**) Baseline medullary tissue oxygen tension (PO₂); (**b**) Comparison of baseline vs. post-AA infusion medullary PO₂; (**c**) Changes in medullary PO₂ relative to baseline; (**d**) Baseline cortical PO₂; (**e**) Comparison of baseline vs. post-AA infusion cortical PO₂; (**f**) Changes in cortical PO₂ relative to baseline. Sample sizes for medullary and cortical PO₂ are shown in panels a and d, respectively, as some data were unavailable due to equipment failure or test subject loss. P-values were obtained using a mixed-effects model with a Greenhouse–Geisser correction for the main effect of time (a, c, d, and f), or by performing multiple paired t-tests with Holm-Šidák correction (**b** and **d**). Data are presented as mean ± SD. * P < 0.05. AA, amino acids; CPB, cardiopulmonary bypass

Compared with pre-CPB baseline levels, both medullary (33.9 ± 9.0 before CPB and 15.1 ± 13.2 mmHg at 4 weeks, P = 0.0068; Fig. 3a) and cortical PO_2_ (46.0 14.2 before CPB and 17.2 ± 6.5 mmHg at 4 weeks, P = 0.0029, Fig. 3d) showed significant and sustained decreases from 1 to 4 weeks after CPB.

The response of medullary PO₂ to AA infusion changed significantly over time (P = 0.0064; Fig. 3b, c). While medullary PO₂ did not change in response to AA infusion pre-CPB and at one week after CPB, it appeared to fall from two weeks thereafter. This reduction was most apparent at two weeks (P = 0.039), showed a tendency toward reduction at three weeks (P = 0.091), but was not observed at four weeks (P = 0.40). In contrast, the response of cortical PO₂ to AA infusion remained unchanged over time (P = 0.43) (Fig. 3e, f).

### Effects of AA infusion on GFR

**Fig. 4:**
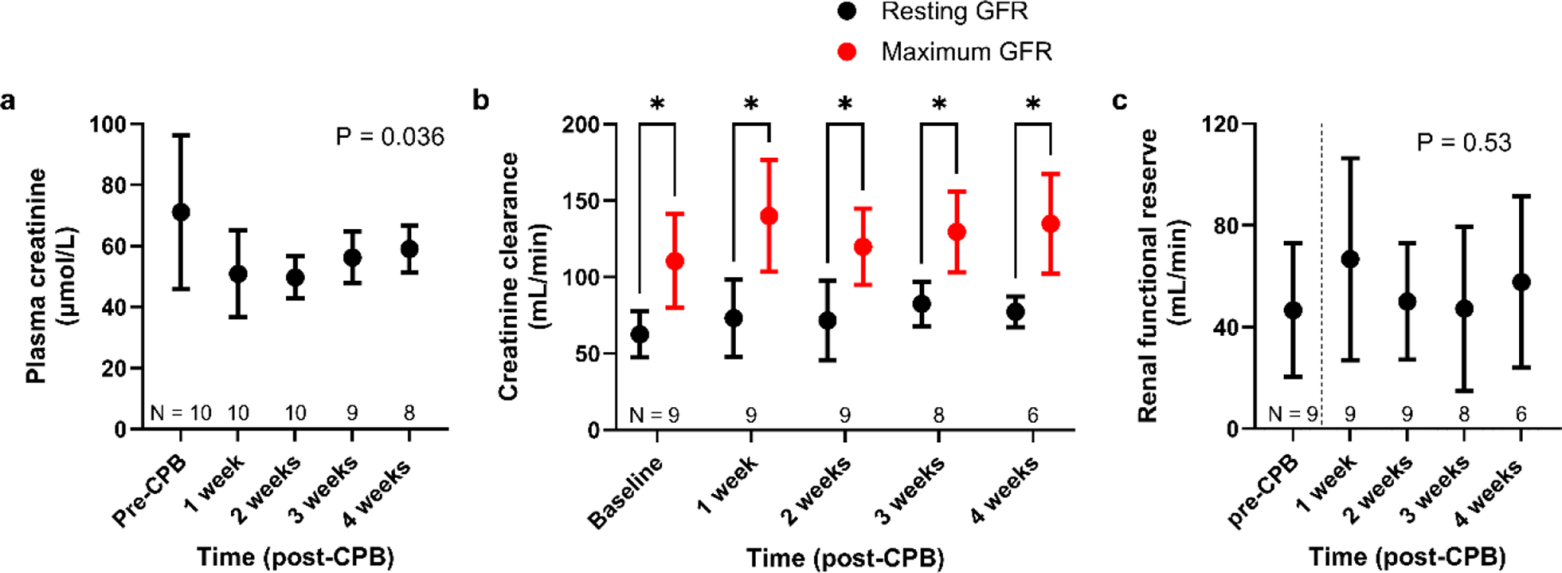
Renal functional indices. **a** Changes in baseline plasma creatinine pre- and post-CPB. Sample sizes are indicated in each panel, as some data were unavailable due to equipment failure or test subject loss. P-values were obtained using a mixed-effects model with a Greenhouse–Geisser correction applied to the main effect of time. **b** Resting (baseline) and maximum glomerular filtration rates (GFR) recruited by amino acid infusion. P-values were obtained using a paired t-test (*P < 0.05). **c** Changes in renal functional reserve, calculated as maximum minus resting GFR. P-values were obtained using a mixed-effects model with a Greenhouse–Geisser correction applied to the main effect of time. All data are presented as mean ± SD. Abbreviation: CPB, cardiopulmonary bypass; GFR, glomerular filtration rates

Baseline plasma creatinine significantly decreased after CPB but gradually returned towards pre-CPB values by weeks 3 and 4 (P = 0.036, Fig. 4a). The administration of an AA load led to a significant increase in urine output (Figure S3). Additionally, it consistently nearly doubled creatinine clearance, indicating successful recruitment of RFR at all time points (Fig. 4b). Before CPB, RFR (maximum minus resting GFR) was 46.7 ± 26.2 mL/min and it did not change significantly over time (*P* = 0.53; Fig. 3c).

### Renal histopathology

**Fig. 5:**
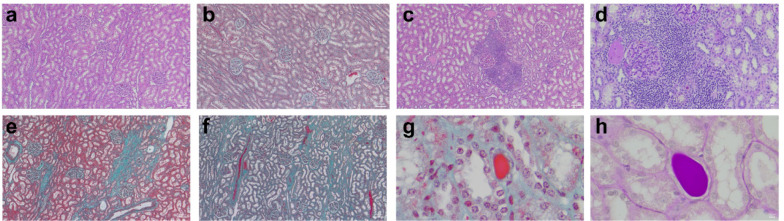
Representative images of histological analysis. (**a**, **b**) Normal kidney tissue; (**c**, **d**) moderate peritubular inflammation; (**e**, **f**) moderate interstitial fibrosis; (**g**, **h**) hyaline casts. White scale bars represent 100 µm in panels a, b, c, e, and f, and 10 µm in all other panels

The histological findings are summarised in Table 1 and Fig. 5. Sheep exposed to CPB showed a significantly greater prevalence and severity of peritubular inflammation compared with healthy controls (8/8 vs. 1/5, *P* = 0.007; severity score: 1 [1–1] vs. 0 [0–0.5], P = 0.0062; Table 1 and Fig. 5c, d). Interstitial fibrosis was more common and severe in the CPB group (6/8 vs. 0/5, P = 0.021; severity score: 1 [0.3–2] vs. 0 [0–0], P = 0.021; Table 1 and Fig. 5e, f). Additionally, hyaline and cellular tubular casts were observed more frequently in the CPB group than in control sheep (8/8 vs. 1/5, P = 0.007; severity score: 1 [1–2]vs. 0 [0–0.5], *P* = 0.0054; Table 1 and Fig. 5g, h) [1]. No changes in glomerular structure or cellularity were observed in either group.

**Table 1:** Renal histological changes in the CPB (n = 8) and healthy control groups (n = 5)

Histopathology	Cardiopulmonary bypass with a 4-week recovery								Control				
	R1	R2	R3	R4	R5	R6	R7	R8	C1	C2	C3	C4	C5
Acute tubular necrosis	0	0	0	0	0	0	0	0	0	0	0	0	0
Peritubular inflammation	+	+	+	+	+ +	+	+	+	0	0	+	0	0
Tubular casts	+	+	+	+	+ +	+ +	+	+	0	+	0	0	0
Interstitial fibrosis	+	+	0	+	+	+ +	+ +	0	0	0	0	0	0
Glomerular changes	0	0	0	0	0	0	0	0	0	0	0	0	0

Histological findings were graded semi-quantitatively as follows: 0, no detectable abnormalities; + , mild changes; + + , moderate changes; + + + = severe changes. This grading was applied to renal tubular injury, peritubular inflammation, tubular casts, interstitial fibrosis, and glomerular changes. The histological analysis in this study was conducted using the same cohort and scoring methodology as the previously published main study [1], with the addition of an assessment of glomerular changes

## Discussion

We conducted a study to measure RFR recruited via an AA load over the 4 weeks following CPB in non-anaesthetised sheep. Additionally, we tested whether exposure to such an AA load had differential effects on medullary and cortical PO_2_ during the post-CPB period, compared with pre-CPB. We found that before CPB, AA infusion increased RBF, induced renal vasodilation, increased oxygen delivery, and successfully recruited RFR, with a near doubling in creatinine clearance. These effects of AA administration persisted after CPB and were consistent throughout the 4-week post-CPB period despite the development of histological injury. We also observed persistent reductions in medullary and cortical tissue PO_2_ after CPB. Furthermore, we found that, while the administration of AA had a similar effect on cortical PO_2_ from pre-CPB throughout each week thereafter, it had a different effect on medullary tissue PO_2_. Specifically, AA infusion did not change medullary tissue PO_2_ before CPB and in the first week, but led to decreased medullary tissue PO_2_ from 2 weeks post-CPB.

Before CPB, we observed marked increases in GFR, urine output, renal blood flow, renal vascular conductance and renal oxygen delivery in response to the administration of AAs. These findings are consistent with previous studies in healthy non-anaesthetised sheep [16]. Importantly, this study extended these observations with serial assessments of these parameters up to 4 weeks after CPB and confirmed that the ability of an AA load to recruit RFR remains after CPB in healthy animals.

In patients with normal preoperative eGFR undergoing elective cardiac surgery with CPB, preoperative RFR, recruited by an oral protein load, has been reported to be a strong predictor of postoperative AKI [20]. Furthermore, long-term follow-up from the same study found that patients who developed post-CPB AKI, or had elevated postoperative cell cycle arrest biomarkers, exhibited a persistent decrease in RFR for at 3 months, despite normalised serum creatinine [9].To date, however, there has been no available assessment of temporal changes in the RFR response to AA in conjunction with observations of renal macro- and microcirculatory function and histopathology following CPB, assessments that can only be conducted in experimental animals.

In our study, AA infusion consistently and markedly increased GFR, RBF, and renal oxygen delivery pre- and post-CPB, without inducing renal tissue hypoxia pre-CPB and at 1 week post-CPB. These post-CPB findings may explain the renal protective effects of prophylactic AA infusion in cardiac surgery as shown in a recent large multicentre RCT [21].Similarly, our findings align with the reported association between a perioperative high-oral protein intake and lower GFR loss at 3 and 12 months in patients with and without AKI after cardiac surgery [22]. Our findings, however, were observed in otherwise healthy animals and in the presence of overt histopathological injury, suggesting a dissociation between renal structural injury and functional assessment based on current clinical biomarkers.

As previously reported [1, 23], medullary PO_2_ appears to decrease post-CPB, and we observed a further decrease in medullary PO_2_ in response to AA infusion from 2 weeks after CPB. This delayed reduction in medullary PO_2_ in response to AA infusion has not been described previously.

Our findings imply that, in healthy experimental animals, recruiting RFR via the administration of AAs is feasible, leads to marked increases in GFR, and persists in magnitude up to four weeks after CPB. This persistent elevation in GFR with AAs may indicate the preservation of RFR after CPB in healthy animals or may reflect a protective effect of repeated weekly AA loading. The preservation of RFR could also be attributed to the relatively mild and focal histopathological changes induced by CPB, which were predominantly limited to the renal tubules and interstitium, with glomerular structures generally well-preserved. However, despite preserved RFR, our findings also suggest that the kidneys exhibit progressive tissue hypoxia. The renal medulla, in particular, appears to be more susceptible to relative hypoxia when challenged during RFR recruitment after CPB. Finally, our observation of preserved RFR in the presence of histological injury suggests that, similar to estimated GFR, RFR may have limited sensitivity for detecting structural injury, and that dynamic function can be preserved even in the presence of structural injury.

Our study has several strengths. We evaluated the temporal changes in RFR, along with the renal microcirculation, renal and systemic haemodynamics, and kidney function over a four-week recovery period following CPB. To our knowledge, this is the first study to examine these changes over such an extended period. Our large mammalian model replicates all key aspects of human CPB, including pump flow, target perfusion pressure, and temperature management, enhancing its relevance to clinical practice [15, 24]. The intra- and postoperative management was collaboratively designed and implemented with cardiac surgeons, cardiac anaesthesiologists, clinical perfusionists, and intensivists to minimise deviations from clinical practice. Importantly, we assessed RFR and the effects of AA infusions in the conscious state, avoiding the potentially confounding influences of general anaesthesia.

We acknowledge some limitations. First, we studied young healthy sheep, which may explain the absence of AKI based on an increase in creatinine criteria, and the lack of changes in RFR. Most patients undergoing CPB have important comorbidities such as hypertension, diabetes, or chronic kidney disease. Therefore, further investigation in comorbid animal models is needed. Second, we only used female animals because urethral bladder catheterization is technically unfeasible in male sheep, and handling larger, more aggressive rams poses occupational health and safety risks. Third, in our model, we accessed the heart via a lateral thoracotomy because the dorsal recumbency position for median sternotomy compromises systemic and renal haemodynamics in sheep [25]. Forth, although we have previously shown that the fibre optic probe causes minimal tissue damage and little fibrosis in both the renal cortex and medulla up to 8 days post-implantation relative to the area of oxygen tension measurement [17], we have not validated the accuracy of the probes beyond this period. Nevertheless, the consistent dynamic changes in response to amino acid infusion at multiple timepoints suggest the probes continued to function during the entire measurement period as intended. Furthermore, the uncoupling of the renal macro- and micro-circulation agrees with our previous findings that whole-kidney oxygen delivery is a poor indication of regional-kidney tissue oxygen tension in ovine sepsis [26, 27]. Finally, due to the small sample size, it is not possible to conduct additional meaningful analysis to explore the relationship between RFR and histological injury in greater detail.

## Conclusions

In a non-anaesthetised large mammalian model of CPB with a 1-month recovery period, we found that the administration of AAs was feasible, easy to execute, and consistently induced marked increases in renal blood flow, renal oxygen delivery and creatinine clearance, indicating successful recruitment of RFR at all timepoints. However, while the AA loading did not change cortical PO_2_ significantly, it led to a reduction in medullary PO_2_ after the second week post-CPB. Despite preserved RFR, there was strong evidence of histological injury at 4 weeks. These findings expand our understanding of post-CPB changes in renal function, oxygenation and tissue injury. They imply that the kidneys maintain a functional capacity to respond to AA loading and support the potential role of such intervention to achieve an increase in GFR after CPB. However, they also highlight the complex relationship between baseline function, RFR recruitment, medullary oxygenation, and structural injury.

## Supplementary Information

Supplementary file 1.

## Data Availability

The datasets used and/or analysed during the current study are available from the corresponding author on reasonable request.
