# Supplementary material for: Recruitment of renal functional reserve by intravenous amino acid loading in a sheep model of cardiopulmonary bypass
Source: Intensive Care Med Exp. 2025 Jul 10;13:71. doi: 10.1186/s40635-025-00774-4 (PMC12246334; doi:10.1186/s40635-025-00774-4)
Supplement: Supplementary file 1 [file 40635_2025_774_MOESM1_ESM.docx]

***Supplemental Material***

**Recruitment of renal functional reserve by intravenous amino acid loading in a sheep model of cardiopulmonary bypass**

Taku Furukawa, MD^1^, Alemayehu H Jufar, PhD^1^, Clive N May, PhD^1,2^, Roger G Evans, PhD^1,3^, Andrew D Cochrane, MBBS^1^, Bruno Marino^4†^, Peter R McCall, MBBS^5^, Sally G Hood, BSc (Hons.)^1^, Ian E Birchall, PhD^1^, Jaishankar Raman, MBBS, PhD^6^, Pei Chen Connie Ow, PhD^1^, Anton Trask-Marino^1^, Lachlan F Miles, MBBS, PhD^1,2,5^, Rinaldo Bellomo, MD, PhD^2,7,8,9†^, Yugeesh R Lankadeva, PhD*^1,2,5^

^1^ Preclinical Critical Care Unit, Florey Institute of Neuroscience and Mental Health, University of Melbourne, Melbourne, Australia.

^2^ Department of Critical Care, Melbourne Medical School, University of Melbourne, Australia.

^3^ Cardiovascular Disease Program, Biomedicine Discovery Institute and Department of Physiology, Monash University, Melbourne, Australia.

^4^ Cellsaving and Perfusion Resources, Melbourne, Australia.

^5^ Department of Anaesthesia, Austin Health, Heidelberg, Australia.

^6^ Faculty of Medicine, Dentistry and Health Sciences, University of Melbourne, Melbourne, Australia.

^7^ Department of Intensive Care, Austin Health, Heidelberg, Australia.

^8^ Australian and New Zealand Intensive Care Research Centre, Monash University, Melbourne, Australia.

^9^ Department of Intensive Care, Royal Melbourne Hospital, Melbourne, Australia.

**Supplemental Figures**


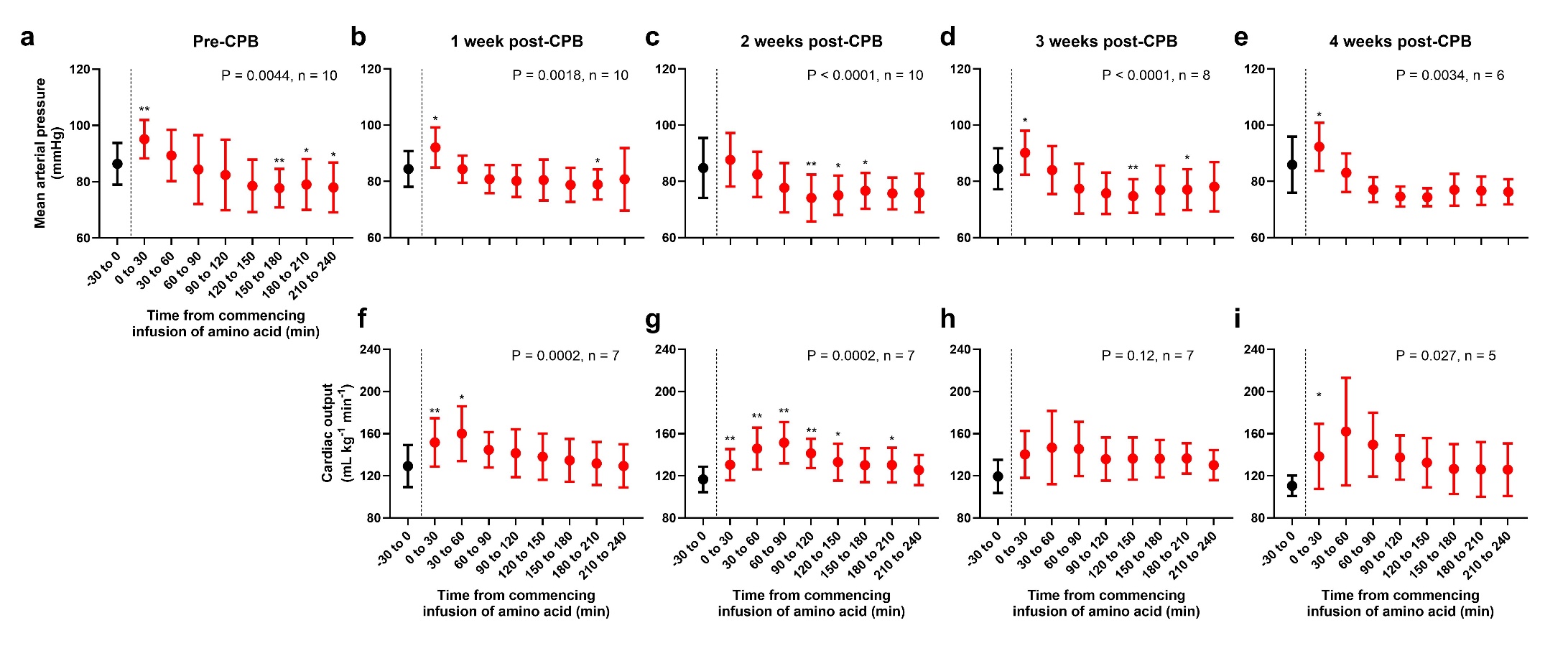


**Figure S1.** Changes in systemic haemodynamics following amino acid infusion. (a–e) Mean arterial pressure; (f–i) Cardiac output. Amino acids were infused over the 0–30 min period. Sample sizes are indicated in each panel, as some data were unavailable due to equipment failure or test subject loss. Cardiac output data were only available post-CPB, as the pulmonary artery flow probe was placed during the CPB experiment. P-values were obtained using a mixed-effects model with a Greenhouse–Geisser correction applied to the main effect of time. When P < 0.05, multiple comparisons were performed using Dunnett’s test against baseline (-30 to 0 min) values (*P < 0.05, **P < 0.01). Data are presented as mean ± SD. Abbreviation: CPB, cardiopulmonary bypass.


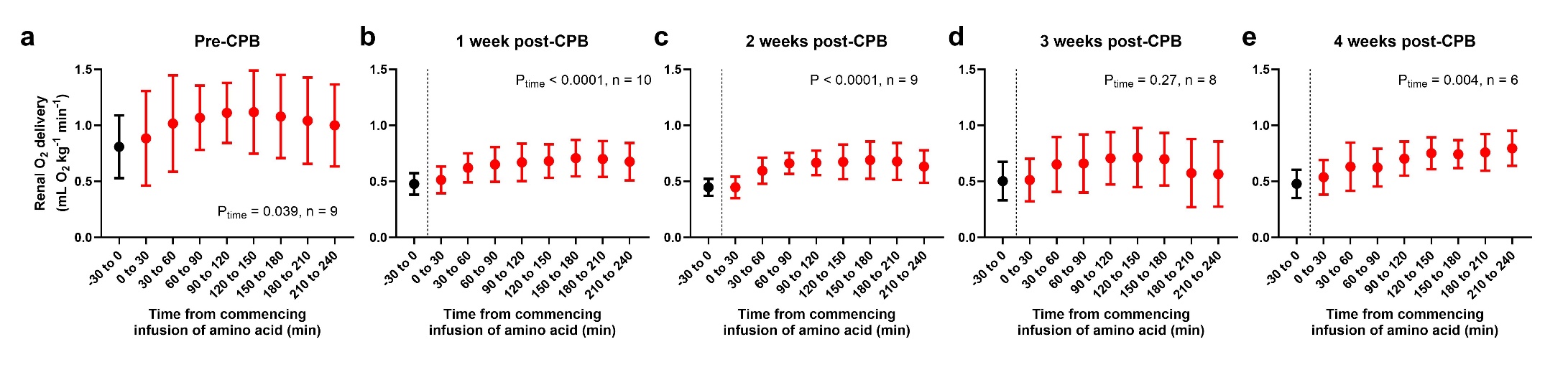


**Figure S2.** Changes in renal oxygen delivery following amino acid infusion. Amino acids were infused over the 0–30 min period. Sample sizes are indicated in each panel, as some data were unavailable due to equipment failure or test subject loss. P-values were obtained using a mixed-effects model with a Greenhouse–Geisser correction applied to the main effect of time. Data are presented as mean ± SD. Abbreviation: CPB, cardiopulmonary bypass.

**
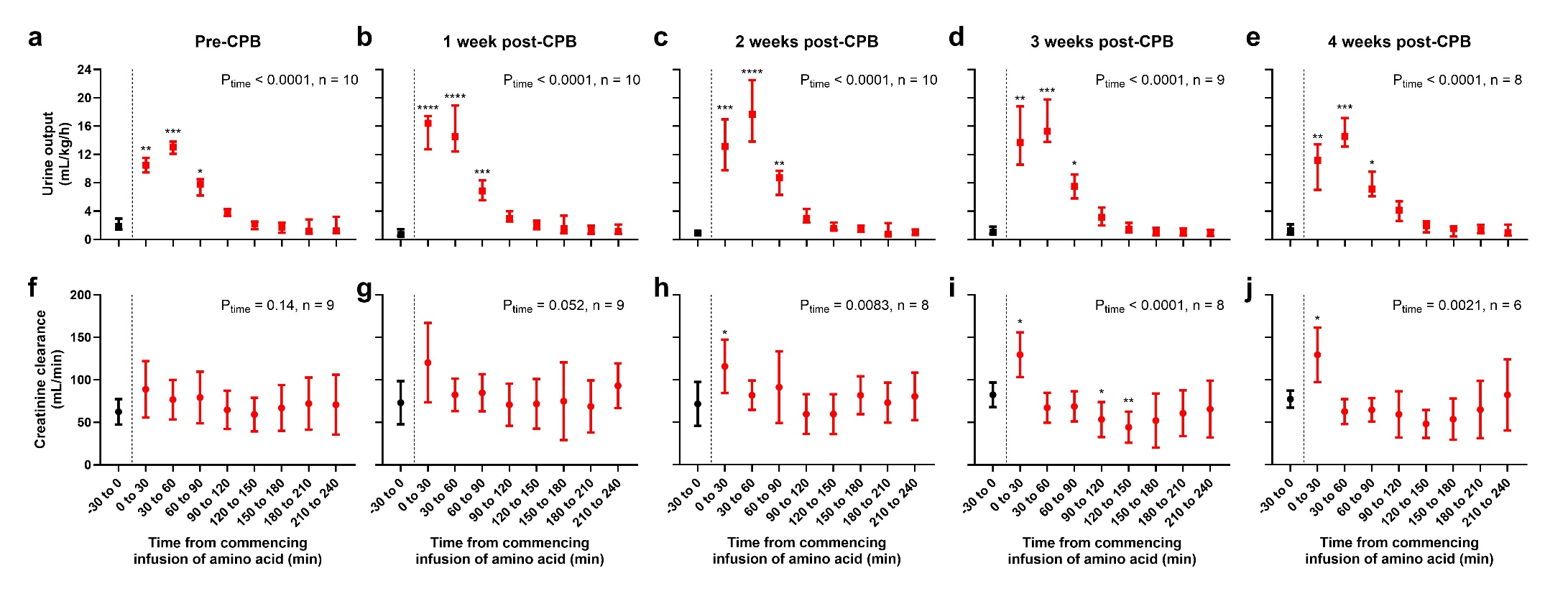
**

**Figure S3.** Changes in urine output and creatinine clearance following amino acid infusion. (a–e) Urine output; (f–j) Creatinine clearance. Urine output data are presented as median and interquartile ranges (squares with error bars), while creatinine clearance data are shown as mean ± SD (circles with error bars). Sample sizes are indicated in each panel, as some data were unavailable due to equipment failure or test subject loss. For urine output, P-values were obtained using Friedman’s test, followed by multiple comparisons against baseline (-30 to 0 min) values with Dunn’s correction if P < 0.05. For creatinine clearance, P-values were obtained using a mixed-effects model with a Greenhouse–Geisser correction applied to the main effect of time, followed by multiple comparisons with Dunnett’s correction if P < 0.05. *P < 0.05, **P < 0.01, ***P < 0.001. Abbreviations: CPB, cardiopulmonary bypass.
